# Mesenchymal stromal cells for osteonecrosis

**DOI:** 10.1186/s12967-020-02565-9

**Published:** 2020-10-20

**Authors:** S. Elgaz, H. Bonig, P. Bader

**Affiliations:** 1 Department for Children and Adolescents, Division for Stem Cell Transplantation and Immunology, University Hospital Frankfurt, Theodor-Stern-Kai 7, 60590 Frankfurt am Main, Germany; 2 Institute of Transfusion Medicine and Immunohematology, and German Red Cross Blood Center Baden-Württemberg-Hessen, Goethe University, Frankfurt am Main, Germany

**Keywords:** Mesenchymal stromal cells (MSC), Osteonecrosis, Cell-based therapies

## Abstract

Osteonecrosis (ON) is an acquired debilitating skeletal disorder, which is caused by a multitude of traumatic and non-traumatic etiological factors. Vascular damage, mechanical stress and increased intraosseous pressure have been discussed as contributors to ON. The optimal treatment of ON remains to be determined, since the current gold standard, core decompression, is insufficiently effective. Specific properties of mesenchymal stromal cells (MSCs) provide the rationale for their assessment in advanced stages of ON: Osteoinductive potential has been demonstrated and MSC preparations of suitable quality for use as medicinal products have been developed. Here we review the scant information on the use of allogeneic or autologous MSCs in advanced ON as well as potentially supportive data from pre-clinical studies with autologous bone marrow mononuclear cells (auto BM-MNCs), which have been studied quite extensively and the presumed therapeutic effect of which was attributed to the rare MSCs contained in these cell products. Outcomes in clinical trials with MSCs and auto-BM-MNCs remain preliminary and non-definitive, at best promising, with respect to their pharmacological effect. Clearly, though, the application of any of these cell therapies was technically feasible and safe in that it was associated with low complication rates. The heterogeneity of cell type and source, study protocols, cell manufacturing, cell properties, cell doses and surgical techniques might contribute to inconsistent results.

## Background

Osteonecrosis (ON), also known as avascular necrosis, is a multifactorial bone disorder that is defined to be debilitating, progressive and refractory [1]. The lesions are prone to progression with collapse of mechanically encumbered subchondral bone and secondary osteoarthritis [2]. Numerous conditions and therapeutic interventions have been associated with the development of ON. Direct damage to bone vasculature, bone or marrow elements is possible. However, the precise pathological mechanism leading to osteonecrosis is not fully understood. Impairment of bone perfusion in traumatic and non-traumatic condition results in the death of bone and marrow cells and subsequent mechanical failure [3]. Reports concerning risk factors for ON largely originate from observational studies [4]. Causes include vascular compromise due to direct trauma, intravascular occlusion in the case of sickle cell aggregations, clots and lipid thrombi, intraosseous extravascular compression due to lipid deposition and adipocyte hypertrophy in the marrow space often associated with corticosteroids or alcohol abuse. Other etiologies have also been related to the development of ON e.g. genetic factors, hyperlipidemia, hyperuricemia, Gaucher’s disease, leukemia and lymphoma [4]. ON is a frequent, and frequently debilitating, late adverse effect of acute lymphoblastic leukemia (ALL) therapy in children and adolescents. The incidence varies between 0.43–17.6% in different studies [5-8]. The application of corticosteroids and other medications such as asparaginase and methotrexate contributes to the pathogenesis, as indicated by the increased incidence in such patients [9]. Correlations between hematopoietic stem transplantation (in case of total body irradiation and chronic graft-versus-host disease (GvHD)) and the development of ON are previously described [10].

ON most frequently develops in the femoral head, likely due to its particularly precarious blood supply; it may also affect the knee, shoulder, elbow and ankle. Where it affects long bones, it occurs predominantly in epiphyses or metaphyses, not infrequently bilaterally. Primarily, the type of treatment should be performed according to presumed etiology. The main therapeutic approaches contain nonsurgical modalities (e.g. anticoagulants, bisphosphonates, statins, vasodilators, hyperbaric oxygenation, extracorporeal shock wave therapy, (single) pulsed electromagnetic fields), joint preserving procedures and joint replacement [11].

Over the last three decades, increasing level of interest in the application of cell-based therapies in the treatment of ON has been noted. Compared with conventional core decompression (CD) alone improved femoral head survival in recipients of cell-based therapies has been reported [1, 12-14]. The apparently pathogenically distinct entity of bisphosphonate-related osteonecrosis of the jaw **(**BRONJ) is explicitly not considered in this review, which focusses instead on the avascular ON associated with acute leukemia and hematopoietic stem cell transplantation. Herein, we summarize and weight the clinical evidence for application of MSCs in the treatment of ON.

## Pathogenesis of ON

Several contributors such as neural, humoral and hormonal factors influence the blood supply to the bone. The interruption of blood circulation leads to ischemia of the dependent area, resulting in demineralization and trabecular thinning. Local ischemia may arise from trauma (fracture) or microtrauma [15]. Two main variants, local and systemic ON are distinguished. Local ON is the consequence of focal trauma, while systemic ON is characterized by multifocal epiphyseal necrosis or bone infarction and is due to more global events [15].

The ultimate pathogenesis of ON remains an area of controversy. Two basic principles concerning the pathogenesis have been proposed: hypoperfusion of lamellar bone and direct injury of osteocytes and osteoblasts [16]. The causes of hypoperfusion comprise vascular malformation, vasculitis, coagulapathies, hemoglobinopathies, myeloproliferative disorders, air embolism, decompression disease, and trauma. Direct toxic damage arises from alcohol or corticosteroid intake, lipid disorders or specific chemotherapeutic agents.

Fundamentally, a distinction is made between intravascular embolism, increased intraosseous pressure and direct blood vessel injury [10, 15]. Circulating lipids, sickle cells, nitrogen bubbles or focal clotting induce microemboli and intravascular coagulation. This results in cell damage and bone marrow edema. Extraluminal obliteration is a consequence of elevated intraosseous pressure, intramedullary bleeding or lipid accumulation, nitrogen bubbles (caisson disease), osteocyte lipid hypertrophy and proliferation of histiocytes in overload disorders (Gaucher’s disease) [10, 15]. The inelasticity of bone results in intraosseous compartment syndrome, reduced blood flow in the bone marrow and disturbance of bone hemostasis. Additionally, damage in the endothelial and smooth muscle cells of supplying blood vessels promote stasis and ischemia [10].

Increased apoptosis of osteocytes, lipid accumulation in osteoblasts and osteocytes lead to deficient bone repair [17, 18]. In this setting, osteoblast differentiation from mesenchymal progenitor cells is also disturbed. Rather than the necrosis itself, the repair process and particularly the resorptive component lead to structural disintegrity and subchondral fracture [4].

The microenvironment, i.e. bone cells (osteoblasts, osteocytes), bone-resident hematopoietic cells, especially specialized macrophage populations, blood vessels, pericytes and endothelial cells, extracellular matrix and mediators secreted by the cocktail of these cells, in ON is disturbed in many respects; while the primary insult appears to be vascular, the resulting ischemia appears to be the dominant cause of all subsequent alterations of bone cells and their microenvironment. Restoration of oxygenation appears to be able to revert much of the damage. Osteonecrotic bone is a severely hypoxic tissue [19]. Underperfusion and, correspondingly, hypoxia are confirmed by metabolic analyses of synovial fluid of ON in canines, where low glucose and high lactate were found, evidencing predominance of anaerobic glycolysis [20]. In pre-clinical studies, direct evidence of hypoxia and of hypoxia response genes was provided. While ischemia is a consequence of an up-stream vascular pathology, the cell death associated with ON is likely a significant part due to hypoxia [21]. Ciapetti et al. showed that proliferation and colony-forming ability were enhanced in hypoxia-exposed MSCs; besides, the expression of alkaline phosphatase, Type I collagen and osteocalcin was increased under hypoxia [19]. These findings suggest that MSCs of patients with ON (ON MSCs) can proliferate and differentiate in the hypoxic area. Adipogenesis seems to be intensified in ON MSCs [22]. Cellular mechanisms of protection against lipotoxicity, such as stearyl-coenzyme A desaturase 1 and carnitine palmitoyl transferase 1 expression are dysregulated in ON MSC; moreover, palmitate-induced interleukin (IL)-6 and IL-8 secretion is higher in ON MSC [22]. Dissection of the Wnt pathways showed higher GSK3ß expression in ON MSC. Other mediators of the Wnt signaling pathway, including runx2 und ß-catenin, exhibit downregulation in ON MSC [23]. Emerging evidence suggests that acquired pathological changes in the bone microenvironment trigger or exacerbate ON. Specifically, cell therapy with MSCs may contribute to their correction by promoting angiogenesis, thus improving oxygen supply to bone and bone marrow, one of the proposed causal pathomechanisms of ON.

In case of trauma, fracture or dislocation can damage extraosseous blood vessels, resulting in ischemia and bone necrosis of the adjacent area. In patients with ALL, leukemia itself may contribute to ON formation due to bone-resorbing effects of lymphoblasts [10]. Evidence for genetic factors in some forms of ON has been reported, e.g. protective effect of multidrug resistance gene in renal transplant patients or ADH2*1 allele of hepatic alcohol dehydrogenase in alcohol abuse [24]. Collagen gene variants were identified in hereditary ON of the femoral head indicating a certain relationship of this entity with the collagen deficiency syndrome osteogenesis imperfecta [15].

ON usually is detected 1–6 months after exposure to a risk factor. If located in proximity to joints, ON predisposes to subchondral fracture with collapse of the necrotic segment of the epiphysis; arthritis necessitating joint replacement often ensues [15]. The correlation between triglyceride levels and risk of ON, demonstrated by studies with lipid-lowering agents, underscores the imputed role of fatty degeneration of bone vasculature in ON [25]. Wang et al. presented in a rabbit model that in steroid-induced ON of the femoral head the adipose differentiation of MSCs was promoted at the expense of osteogenic differentiation [26].

## Biological properties and effects of MSCs in bone regeneration

MSCs represent a heterogeneous population of multipotent progenitor cells with variable proliferative, differentiation, immunoregulatory and immunosuppressive potential and which are capable of forming colony-forming fibroblasts [27, 28]. In the end of 1960s, Alexander Friedenstein and colleagues’ pioneering work discovered MSCs, since then these cells have attracted considerable scientific interest [29]. In 2006, the International Society for Cellular Therapy (ISCT) published a position paper concerning minimal criteria for defining multipotent mesenchymal stromal cells [30]. The following criteria were determined at the time: plastic adherence; expression of CD105, CD73, negativity for CD45, CD34, CD14, CD11b, CD19, HLA-DR, and trilineage differentiation potential in osteoblasts, adipocytes and chondroblasts. Of note, this is the phenotype of fetal calf serum-expanded MSCs in two-dimensional cultures; recent advances in MSC propagation challenge several of these criteria. Besides this, MSCs show a high propensity for ex vivo expansion, which facilitates their ex vivo generation for medicinal purposes. In the beginning, MSCs were isolated from bone marrow, subsequently also from other tissues. They have been identified in postnatal and adult tissues; their frequency declines with age [31].

As therapeutic cells in vivo, MSCs show anti-inflammatory and regenerative effects by secretion of molecules [32]. Uniquely for a medicinal product, MSCs apparently respond to damage signals with secretion of tissue-specific factors, e.g. responds to inflammation with anti-inflammatory mediators, to vascular damage with angiogenic factors, etc. In ON one would envision therapeutic benefits from signals supporting vascular repair, bone regeneration or protection of bone cells from cell death. The aforementioned concepts are the very high-level summary of a vast body of experimental research, which has provided the rationale for clinical translation. Thus, numerous clinical trials are currently ongoing for MSCs or MSC-containing cell preparations. The application of MSCs or MSC-containing cell preparations has been accomplished in cardiovascular disease, bone regeneration and GvHD after hematopoietic stem cell transplantation [14, 33-36]. The furthest advanced towards routine clinical application is treatment of therapy refractory acute GvHD. In a large number of clinical trials or case series MSCs have been investigated as novel cellular therapy in acute GvHD [34, 37-44]. Encouraging results in this field up to over 80% overall response rate have been reported [37, 45].

It is furthermore known that MSCs regulate osteogenesis and osteclastogenesis by the release of multiple soluble factors such as cytokines (interleukin (IL)-1β, IL-6, IL-11, osteoprotegerin (OPG)), chemokines (RANKL), growth factors (LIF, FGF-2, M-CSF, PDGF, TGF- β) and others (PGE2, DKK-1, Wnt 2,4,5,11,16) [31]. Autocrine and/or paracrine pathways regulate the differentiation of osteoclasts. Osteoblasts also influence the myelopoietic and hematopoietic process by releasing multiple factors. A recent study conducted by Abe et al. revealed that mouse bone marrow-derived MSCs suppress osteoclast differentiation by inhibiting the expression of receptor activator of NF-κB (RANK), colony stimulating factor 1 receptor (CSF1R), NF-κB and nuclear factor of activated T-cell cytoplasmic 1 (NFATc1); whereas the expression of OPG appeared to be involved in the inhibition of osteoclast differentiation [46].

The predicted capacity of MSCs for in vivo bone formation was initially confirmed in the pioneering study of Horwitz et al. [47]. Six children with severe osteogenesis imperfecta (OI) underwent allogeneic bone marrow transplantation. Each patient received two infusions of donor-derived MSCs (total dose 2–6 × 10^6^ /kg). Engraftment in one or more sites could be demonstrated in five of six patients, in whom acceleration of growth velocity was observed in the first six months after infusion. The presented data indicate that allogeneic MSCs can engraft in genetically defective bone and contribute to new bone (matrix) formation, although whether the few engrafting MSCs were derived from the BM graft or the co-administered culture-expanded isogeneic MSCs could not be answered. In 2005, Le Blanc et al. reported a case of a female fetus with multiple intrauterine fractures, diagnosed as severe OI. In the 32^nd^ week of gestation, 3 weeks prior to delivery, the fetus underwent allogeneic HLA-mismatched male fetal MSCs transplantation (6.5 × 10^6^ cells). At nine months of age, donor cell contribution to bone biopsies exhibited, on average, 7.4% whole Y genome fluorescent in situ hybridization (FISH) staining [48]. What these two reports have in common is that MSCs appear to have differentiated into bone cells, engrafted and produced bone cell-typical extracellular matrix, but also that MSCs were transplanted into a milieu which is rarefied for endogenous bone cells. Thus, in analogy to severe combined immunodeficiencies where donor immune cells enjoy a selective growth advantage, MSCs may have found a supportive niche in OI bone.

## Clinical experience with MSCs in the treatment of ON

The imputed therapeutic role, or mechanism, in ON is controversial, the possibilities of osteoinduction/paracrine activities and integration after differentiation into bone or blood vessel cells have both been proposed. A limited number of studies were performed to determine the efficacy of therapeutic cell implantation, not all of them with MSC, into necrotic lesions on clinical symptoms and the progression of ON in comparison with other therapeutic procedures (Table 1).

**Table 1:** Overview of clinical studies on MSC- or auto-BM-MNC therapy in ON

Reference year	Diagnosis	Intervention/ source	Study design	Cohort	Classification	Cell dose	Follow-up	Results
Müller et al. 2008 [14]	ONFH + ON of the knee	CD + autologous BM MSCs	Pilot study	pts = 5	not specified	MSCs 31 × 10^6^–240 × 10^6^	16 mo	Clinical improvement, formation of mineralized bone in the necrotic area
Zhao et al. 2012 [35]	ONFH	CD ± autologous BM MSCs	Single center randomized clinical trial	pts = 100	ARCO IC-IIC	MSCs 2 × 10^6^	60 mo	2 of 53 BMMSC-treated hips progressed; in CD group 10 of 44 hips progressed; significant improvement of HHS in BMMSC group
Chen et al. 2016 [49]	ONFH (steroid, alcohol, idiopathic)	Intra-arterial infusion of human umbilical cord-derived MSCs	Retrospective analysis	pts = 9	ARCO II-IIIa	MSCs 5 × 10^6^–1 × 10^7^	3 y	Significant reduction of the necrotic volume on MRI 12/24 months after application
Hernigou et al. 2009 [54]	ONFH (steroid, alcohol, SCD	CD + reduced volume autologous BM	Single center study	pts = 342 hips = 534	Steinberg I-II	CFUs 24 × 10^3^	8–18 y	Total hip replacement in 94 hips (n = 534); total resolution of ON in 69 hips
Daltro et al. 2015 [58]	ONFH (SCD)	CD + autologous BMMCs	Phase I/II prospective trial	pts = 89	Ficat 0-IIB	CFU-F 2.7 ± 1.4 × 10^4^/cells	60 mo	Significant improvement in HHS (p = 0.0005); 3.7% no satisfactory outcome
Gangji et al. 2011 [55]	ONFH (steroid, alcohol, idiopathic)	CD ± autologous BMMC	Controlled double blind pilot study	pts = 19 hips = 24 BM hips = 13	ARCO I-II	BMMCs 1.9 ± 0.2 × 10^9^: - CD34^+^ 1 ± 0.1% - CFU 92.6 ± 22.4 × 10^7^/cells	60 mo	Significant reduction in pain/ joint symptoms and reduced the incidence of fractural stages; significant difference in time to failure
Sen et al 2012 [56]	ONFH (traumatic, nontraumatic)	CD ± autologous BM concentrate	Randomized control study	pts = 40 Hips = 51	ARCO I-II	BMMCs 5 × 10^8^: CD34 + 5 × 10^7^		Significant clinical improvement in HHS
Martin et al 2013 [53]	ONFH (steroid, alcohol, idiopathic)	Minimally invasive decompression + concentrated BM aspirate	Retrospective review	hips = 77	Ficat I-II	not specified	17 mo	Progression in 16 hips (21%); significant pain relief in 86% of patients (n = 60)
Civinini et al 2012 [59]	ONFH	CD + autologous BM concentrate and backfilling with bioceramic	Prospective single center trial	pts = 31 hips = 37	Steinberg IC-IIIA	not specified	20.6 mo	Improvement in HHS; clinical success rate 86.5%, failure rate 3.3% in pre-collapse group
Pepke et al 2016 [52]	Non-traumatic ONFH	CD ± autologous BM concentrate	Randomized prospective study	pts = 24 hips = 25	ARCO II	BMMCs 118.9 ± 15.1 × 10^6^ cells/ml	24 mo	No difference in clinical outcome and head survival rate between both groups

*ARCO* Association Research Circulation Osseous, *BM* bone marrow, *BMMCs* bone marrow mononuclear cells, *BMMSCs* bone marrow mesenchymal stromal cells, *CD* core decompression, *CFU* colony forming units, *HHS* Harris Hip Score, *MSCs* mesenchymal stromal cells, *ON* osteonecrosis, *ONFH* ON of femoral head, *pts* patients, *SCD* sickle cell disease

We will first discuss the very limited body of evidence for use of culture-expanded MSCs in the treatment of ON. Müller et al. were the first to assess the feasibility and safety of the autologous MSC application during CD in a small patient group (n = 5) [14]. Starting from a 10 ml bone marrow aspiration, MSCs were selected by plastic adherence and expanded for 3 passages in platelet lysate (PL) enriched culture medium, at which point 31–240 million MSCs were harvested and instilled into the ON lesion in a volume of 3 ml. The authors reported feasibility of auto MSC generation, safety and tolerability of the MSCs, and the impression of a clinical benefit as well as formation of mineralized bone in the necrotic area demonstrated by computed tomography [14]. Müller et al. favor a “cytokine factory” type therapeutic mechanism of MSCs in ON. Of potential relevance to the outcome, they tested low-oxygen culture conditions for MSCs, under which vascular endothelial-derived growth factor (VEGF) and insulin-like growth factor binding protein (IGFBPs) were elevated [14].

Zhao et al. followed up on the pioneering work of Müller with a randomized prospective trial comparing CD with autologous BM-MSCs combined with CD alone in ON of the femoral head [35]. The MSCs were mitotically younger, with an ex vivo expansion for only two weeks, and the dose only 1% of that used by Müller et al. 2/53 vs. 10/44 ON lesions in the CD + MSC-treated vs. CD-only-treated progressed; the BMMSC group was also reported to enjoy significant improvement in HHS and decrease in volumetric involvement of femoral head. Thus, a patient-relevant benefit from auto-BM-MSCs is suggested.

MSC from a different source and a different route was applied by Chen and colleagues [49]. They present a retrospective analysis from a series of nine patients with ON of the femoral head which was treated intra-arterially with human umbilical cord-derived MSCs without CD. A dose of 50–100 million MSCs was injected into the right femoral artery under the hypothesis that the cells would be retained in the ON lesion and there differentiate into either vascular or bone cells [49]. At three days post-operation the oxygen delivery index increased, and at 24 months follow-up a significant reduction of the size of the necrotic cavity according to MRI was reported, which the authors attribute to the cell therapy. Direct evidence of cell integration or cell-mediated transient or long-term effects was not provided.

More recently, the rationale for use of MSCs in ON has been externally validated in several animal models of ON which confirmed beneficial effects of local MSCs application [50, 51]. Allogeneic peripheral blood-derived mesenchymal stem cells from rabbits were transplanted into rabbits with ONFH. After local transplantation, increase in bone density and bone trabeculae was observed [50].

While the work provided is insufficient to allow definitive conclusions about clinical efficacy of MSCs in ON the published work agrees on its safety: In none of the patients complications or adverse effects of the medicinal product were encountered [35, 52-54].

In addition to this very limited body of work with culture-expanded MSCs, a succession of studies has tested autologous BM-MNCs. Although the bulk of these cells, in fact, more than 99%, are hematopoietic in nature, most of the authors hypothesize that the therapeutic component in bone repair might lie in the MSC compartment [54-56]. However, a role of hematopoietic cells in ON repair cannot be dismissed. Thus, Henrich et al. demonstrate in a critical size bone defect model in the athymic rat that BM-MNC promote bone repair and that this therapeutic activity is restricted to a CD14 + , i.e. monocyte/macrophage population, since its depletion abrogated the benefit of BM-MNCs [57]. The cumulative experience with auto-BM-MNC in ON is summarized below.

Hernigou and colleagues pioneered the clinical application of auto-BM-MNC grafting and CD for ON of the hip between 1990 and 2000 in a large patient cohort [54]. During this period, 342 patients (534 hips) at early stages ON (Steinberg I-II) were treated. The cell therapy product was generated from autologous BM by removing red blood cells and plasma, thus more than 99% of the cells were blood cells, predominantly mature leukocytes but also containing hematopoietic stem cells and some MSCs. These cells were injected into the femoral head lesion after decompression with a trocar. Ninety-four hips progressed to collapse, necessitating total hip replacement. Total resolution of ON was observed in 69 hips, no control group was investigated. The authors postulated symptomatic hips without collapse as the best indication for their method. Using an essentially similar cell therapy product, Daltro et al. conducted a phase I/II, non-controlled trial in patients with sickle cell disease (SCD) and ON of the femoral head [58]. Only 3.7% of the patients (n = 89) failed to achieve satisfactory clinical outcomes, which were defined as improved pain scores. Measured by Harris Hip Score (HHS), significant pain relief and disease stabilization were reported at the final follow-up after 60 months. In patients with SCD the prevalence of ON is up to 50% with substantial limitation in physical activity. In this study, SCD patients could be treated safely. Disease progression was arrested if treated at early stages, but how much of the outcome is attributable to the cells vs. the CD cannot be answered.

Gangji et al. demonstrated in a controlled double-blind pilot study, also with auto-BM-MNC, with long-term follow-up (60 months), initiated at Erasme Hospital (Bruxelles), a significant reduction in time to failure in the bone marrow graft group as well as significant improvement in symptomatic complaints. Nineteen patients (24 hips) with early stage ON of the femoral head were evaluated in the trial, 13 hips received implantation of auto-BM-MNC. The bone marrow harvest from the iliac crest was concentrated and implanted into the femoral head. The rate of progression to the fractural stage of ON (ARCO stage 3) was significantly reduced in the bone marrow graft group [55].

Sen et al. also analyzed the use of auto-BM-MNC with CD in patients with femoral head ON [56]. Fifty-one ARCO stage I or II osteonecrotic hips in 40 patients were treated in a randomized control trial. Mean HHS in group A (only CD) at the end of 12 months was 76.68, whereas group B (auto-BM-MNC and CD) had a score of 83.65. Mean hip survival was 46.7 weeks in group A and 51.8 weeks in group B, respectively. Both outcomes were significant in favor of auto-BM-MNC (p < 0.05). Notably, pre-operative etiologies affected outcome and patients with adverse prognostic features at initial presentation had significantly better clinical outcome and hip survival in the auto-BM-MNC treated group.

Martin et al. reported minimally invasive decompression technique concomitant to local injection of concentrated bone marrow and platelet rich plasma [53]. The procedure was utilized in 77 hips. Hip replacement was required in 16 progressed hips (21%). Sixty patients (86%) experienced significant pain relief.

Civinini et al. conducted a prospective, single center trial, which included 37 hips (31 patients) that underwent CD with injection of auto–BM-MNC and backfilling with calcium sulphate/calcium bioceramic in the treatment of ON of femoral head [59]. At the final follow-up, the HHS was improved, and the radiological imaging showed improvement in 29 hips (78.4%). This overall clinical success was calculated to be 86.5%; for lack of concurrent controls a possible contribution of the cells vs. CD vs. spontaneous improvement cannot be ascertained.

In their controlled study, Pepke et al. reported no difference in clinical outcomes after additional injection of auto-BM-MNC during CD (52). Twenty-four patients were evaluated in randomized, prospective trial during two years after procedure. Over this period, no significant difference in head survival rate and no significant reduction in the necrotic area volume in both cohorts were observed.

The upshot of these auto-BM-MNC studies appears to be that, while very safe, the added therapeutic benefit over CD alone is at best modest, but also that CD alone does not provide satisfying results.

## Discussion

ON is a debilitating disease associated with the risk of collapse and arthroplasty in younger populations followed by poor joint outcomes. Decreased oxygen supply and impaired osteogenic potential of circulating progenitor cells associated with an inflammatory microenvironment are considered the pathogenic mechanism [60]. Multipotent MSCs have been investigated in numerous clinical trials thus far, albeit few of them in ON. Particularly, immunoregulatory features have been exploited in the clinical applications [34, 37]. With respect to MSCs and bone repair, it is currently unclear, which mechanisms, osteogenetic or osteoinductive, are imputable for therapeutic effects, if any. MSCs are assumed by some to exert osteogenic or angiogenic potential due to their capacity of multilineage differentiation. Supporting this hypothesis, the pioneering study by Horwitz et al. as well as intrauterine transplantation of HLA-mismatched allogeneic MSCs unexpectedly demonstrated their sustained engraftment in bone regeneration [47, 48]. On the other hand, several studies indicate that the therapeutic effect subsequently emerges via paracrine mechanisms [28, 61]. Beneficial effects by inducing cell proliferation and prevention of cell apoptosis make them suitable for bone tissue engineering [62]. The secretion of cytokines and growth factors at site of injury are attributed to biological properties of MSCs [28]. Increasing osteoblasts and capillaries as well as VEGF and BMP-2 expression were observed after MSCs implantation in the necrotic area [63]. Additional research is needed to determine the specific pharmacological mechanisms. Whatever the mechanism, various clinical trials were conducted to assess feasibility and safety as well as efficacy of MSCs or auto-BM-MNCs in the setting of ON treatment.

The published work on MSCs is too preliminary to support claims of efficacy in ON. Reported results with auto-BM-MNCs, where relevant controls were included, similarly do not provide much confidence about a therapeutic benefit. Recently, an excellent review by one of the pioneers of cell therapy for ON has come forth [64]. Hernigou analyzed existing challenges in the future for MSC application in ON. Inconsistencies in clinical outcomes might arise due to different study protocols, patient characteristics, different outcome assessments, lack of standardized cell generation methods and cell dosing [33]. In some studies, small sample sizes (n < 50) or selection bias may also affect the final results. An insightful paper reviewing use of hematopoietic cells for ON treatment was recently published and comes to essentially similar conclusions as us [65]. The utilization of cell therapies in ON demonstrated heterogeneity in the choice of cells, cell processing methods, quantitative and qualitative assessment of the cells, technical and surgical methods and cell delivery. The lack of standardization and broad variation produce general interferences and difficulties in reproducibility. Greater benefit could be obtained from investment in blinded, randomized, controlled trials [65]. In order to establish suitable protocols, the effects of concentration and culture based preparatory methods for MSC therapy should be compared [66].

Taking a more systematic approach than ourselves with this review, Yuan et al. implemented a meta-analysis of seven case–control studies of auto-BM-MNC implantation in the ON treatment of the femoral head [12]. The cell-therapy group revealed improved clinical results with delayed progression (odds ratio (OR) = 0.17; p < 0.001), reduced total hip arthroplasty incidence (OR = 0.3; p < 0.01) and increased HHS (mean difference = 4.76; p < 0.01). Similar results were obtained from another meta-analysis done by Wang et al., which included some of the previous studies (overlap) [67]. However, it should be noted that the comparability of studies is limited because of the large heterogeneity of different methods and patients’ characteristics, which could influence the validity of the analysis.

While we lack strong data to address the clinical relevance of the MSC application in ON, some pre-clinical work offers preliminary insight. Rapp et al. reported the superiority of autologous MSCs in a humanized mouse model compared to allogeneic MSCs [68]. Less bone formation, impaired angiogenesis and reduced expression of osteogenic factor Runx2 were found in mice treated with allogeneic MSCs [68]. Berner et al. showed in an ovine model similar bone regeneration by autologous and allogeneic MSCs and hypothesized the potential for an off-the-shelf product [69]. Several important caveats of auto-MSCs must be considered in informing the choice of allo vs. auto: Allo-MSCs can be produced off-the-shelf and will thus be immediately available, the logistics are also much easier than with auto-MSCs. Possibly more important is the fact that auto-MSCs are generated from patients, thus have been subject to the same agents that caused the ON in the first place. It is therefore not unreasonable to wonder about the pharmaceutical quality of patient-derived MSCs. To this end, Houdek et al. compared the cellular viability and function as well as ability to multilineage differentiation of MSCs from ON patients [70]. MSCs isolated from patients with corticosteroid-induced ON had decreased cellular activity and ability to differentiate in comparison to control MSCs from healthy donors. These findings could be attributed to chronic steroid exposure leading to preferential differentiation of MSCs into adipose or cartilage tissue. We therefore propose using healthy-donor MSCs generated and tested according to robust, dose-to-dose similarity enforcing protocols. Clearly, rigorous randomized prospective interventional trials based on these issues are currently needed.

Some studies further suggest that earlier intervention with CD may be more efficient in preventing progression and limiting functional impairment [1, 35, 53, 55, 58]. Accordingly, ON screening of patients with known risk factors (e.g. leukemia, sickle cell disease, systemic lupus erythematosus, prolonged high doses of steroid intake, alcohol abuse) might be beneficial. Markers of bone matrix generation were shown to be sensitive, even quantitative indicators of therapeutic response to therapy with MSCs and should thus reasonably accompany clinical trials [71]. Transplantation of modified MSCs provides promising efficacy of ON clinical therapy in animal models [72]. Compelling evidence for a role of engineered hepatocyte growth factor secretion by MSCs has been put forth; improved engineered MSC persistence by compounding with fibrin glue was shown to improve clinical efficacy in a rabbit model of ON [72]. Modified MSCs could, as preliminary data suggest, further improve the therapeutic efficacy of MSCs in ON, but will find clinical application only if satisfactory effects cannot be achieved with optimized protocols and primary culture-expanded MSCs.

Meanwhile gene modification techniques to overexpress growth factors and transcription factors have been reviewed in small-animal models in order to enhance angiogenesis and osteogenesis [73]. This approach supports preliminary evidence of efficacy; nevertheless, such therapies remain highly experimental.

## Conclusion

The aim of this systematic review was to evaluate the application of MSCs in ON. In conclusion, although based on preliminary data throughout, the use of MSCs and BMMNCs in osteonecrotic lesions is very safe. Autologous and allogeneic sources of MSCs have been investigated thus far. The published work on MSCs is preliminary, so that the efficacy in ON is non-definitive, but promising. With regard to the data currently available, further research using auto and allo MSCs in ON, including controls in the framework of randomized trials, is required.

## Data Availability

Not applicable.

## Abbreviations

ALL: Acute lymphoblastic leukemia
ARCO: Association Research Circulation Osseous
auto BM-MNCs: Autologous bone marrow mononuclear cells
BM: Bone marrow
BMMSCs: Bone marrow mesenchymal stromal cells
CD: Core decompression
CFU: Colony forming units
FISH: Fluorescent in situ hybridization
HHS: Harris Hip Score
ISCT: International Society for Cellular Therapy
GvHD: Graft-versus-host disease
MSCs: Mesenchymal Stromal Cells
ON: Osteonecrosis
ONFH: ON of femoral head
ON MSCs: MSCs of patients with ON
OPG: Osteoprotegerin
SCD: Sickle cell disease
